# Prevalence of anti-NMDA receptor antibodies among adult patients presenting with first-episode psychosis in Hong Kong

**DOI:** 10.1038/s41537-026-00741-2

**Published:** 2026-02-25

**Authors:** Steven Wai Ho Chau, Charis KW Lam, Matthew PM Yu, Joseph CH Choi, Howan HW Leung

**Affiliations:** 1 Department of Psychiatry, The Chinese University of Hong Kong, Hong Kong, China; 2 Department of Psychiatry, Prince of Wales Hospital, Hong Kong, China; 3 Faculty of Medicine, The Chinese University of Hong Kong, Hong Kong, China; 4 Division of Neurology, Department of Medicine and Therapeutics, Prince of Wales Hospital, Hong Kong, China

**Keywords:** Psychosis, Psychosis

## Abstract

Studies on the presence of anti-NMDA receptor antibodies were done primarily on the European population. This study investigates the prevalence of anti-NMDA antibodies among adult patients presenting with first-onset psychosis in an acute medical setting in the predominantly Chinese population of Hong Kong (*n* = 109), and our results suggest that such prevalence is low in this population (1.8%), especially among patients with primary psychotic disorders (0%). Further studies are needed to explore the ethnic variation in anti-NMDA receptor positivity.

Anti-NMDA receptor antibodies are the most common type of anti-neuronal antibodies. The description of anti-NMDA receptor antibodies among patients with autoimmune encephalitis in 2007^1^ has since stimulated interest in the role of autoimmunity in the pathophysiology of psychotic disorders, as the functional disruption of anti-NMDA receptor antibodies fits well in the NMDA-hypoactivity hypothesis of schizophrenia^2^. Studies from the past decade report the prevalence of anti-NMDA receptor antibodies among patients with primary psychotic disorders, such as schizophrenia, to be around 1–4%, with considerable variability among the studies^3–5^. While the role of anti-NMDA receptor antibodies and other types of anti-neuronal autoantibodies in the pathophysiology of psychiatric disorders in these seropositive patients remains debated^6,7^, evidence suggests that psychotic patients with serum antineuronal antibody positivity (including but not limited to anti-NMDA receptor antibodies) have a different clinical profile from the rest^4,8^, and may benefit from immunotherapy^9^. To accommodate these latest understandings, the concept of autoimmune psychosis has been proposed to carve out a subgroup of psychotic cases with potential autoimmune mechanisms^10^. Although this concept is still fluid and rapidly developing^11^, the identification of this minor but distinct subgroup is potentially paradigm-shifting for treatment for psychotic disorders, and possibly other psychiatric disorders as well.

However, studies on the prevalence of anti-NMDA receptor and other antineuronal autoantibodies among psychotic patients have primarily been conducted in European populations, and data from other populations, such as Asians, are underrepresented. Such disparity limits our understanding of the epidemiology of seropositive/autoimmune psychosis at a worldwide level. The epidemiology of autoimmune phenomena is known to be varied among ethnic groups. For example, multiple sclerosis is more common in the White population compared to the Asian population, while the reverse is true for systemic lupus erythematosus^12^. In addition, most of the existing studies did not collect samples during the acute phase of psychosis. For example, the only available study examining the prevalence of anti-NMDA receptor antibodies among Chinese with first-episode psychosis was done in the setting of a specialist outpatient for early psychosis in Hong Kong^13^, which would have excluded patients with a diagnosis of autoimmune encephalitis.

The primary objective of the current study is to investigate the prevalence of anti-NMDA antibodies among Chinese adult patients presenting with unmedicated first-onset psychosis in an acute medical setting. The secondary objective is to explore the presentation of patients with psychosis and anti-NMDA receptor antibodies. Our sample included 109 patients presenting with first-onset psychotic symptoms from January 2015 to March 2025, who were tested for anti-NMDA receptor antibodies (Mean age = 36.0 years; 71.6% female; 96% ethnic Chinese). Since the selection of patients for antibody testing was based on clinical suspicion of anti-NMDA encephalitis by the treating clinicians, the patients tested had significantly different demographic and clinical characteristics, such as age, acuteness, presence of fever, and catatonia compared to those who were not tested. (See Table 1). Cerebrospinal fluid (CSF) samples were tested in 60 of them. Among the tested patients, 35 (32%) were diagnosed with non-specific psychosis, 21 (19%) were diagnosed with schizophrenia, 15 (14%) were diagnosed with acute and transient psychotic disorder, 10 (9%) were diagnosed with mood disorder with psychotic symptoms and 19 (17%) patients were diagnosed with psychosis secondary to substance use or other medical conditions. Two of the tested patients (1.8%) were found to be positive for anti-NMDA receptor antibodies (ages 20 s to 30 s; 1 female, both are Chinese). The antibodies were detectable from both blood and CSF samples in both patients, and they both fulfilled the diagnostic criteria of definite autoimmune encephalitis suggested by Graus et al. ^14^. The first patient presented with abrupt-onset insomnia, cognitive impairment, and auditory and visual hallucinations within a week. The patient subsequently developed orofacial dyskinesia and seizures in the following two weeks. While the MRI of the brain with contrast did not reveal encephalitic changes, the CSF analysis showed marked pleocytosis without evidence of infection. The second patient presented with an acute onset of cognitive impairment, agitation, and insomnia for two weeks, followed by the onset of auditory hallucination two days before hospitalization. CSF analysis of the patient showed marked pleocytosis without evidence of infection. The MRI brain scan showed an abnormal T2W signal over the left temporal lobe, suggestive of encephalitis, which resolved after treatment. In both patients, extensive work-ups, including full-body PET-CT scans, did not reveal any malignancy. Both patients’ neuropsychiatric symptoms responded to aggressive immunotherapy, which included the sequential use of intravenous immunoglobulin, intravenous high-dose pulse methylprednisolone, plasmapheresis (in one patient), and intravenous rituximab.

**Table 1:** Comparison of demographical and clinical characteristics between patients with first-episode psychosis tested for anti-NMDA receptor antibodies and those untested.

	FEPs without anti NMDA-R ab tested (*n* = 280)	FEPs with anti NMDA-R ab tested (*n* = 109)	*P* value
Mean age(SD), years	41.6(13.5)	36.0(12.8)	<0.001^a^
Female, %	67.9%	71.6%	0.48^b^
Non-ethnic Chinese, %	2.9%	3.7%	0.76^c^
Onset < 1 month, %	27.1%	56.8%	<0.001^b^
Presence of fever (>= 37.5 C), %	20.0%	43.1%	<0.001^b^
Catatonia at presentation, %	1.8%	8.3%	0.004^c^

^a^*t* test was used.

^b^Chi-square test was used.

^c^Fisher’s exact test was used.

To the best of our knowledge, this is the first study investigating the prevalence of anti-NMDA receptor antibodies among patients presenting with first-episode psychosis in an acute medical setting in a predominantly Chinese population. Our results suggest that the prevalence of anti-NMDA receptor antibodies among patients with first-onset psychosis is low among our cohort (1.8%). Particularly, we do not find any antibody-positive case among patients suffering from primary psychotic disorders. A previous study on the prevalence of anti-NMDA receptor antibodies among patients with psychotic disorders in an outpatient setting in Hong Kong Chinese also yielded a low prevalence of 1.5%^13^. The prevalence reported in the literature, predominantly based on the Western population, has been highly variable. This variability can be partly attributed to diverse sampling contexts among the studies. The different kinds of assay used by the study could also explain part of the variability, as live cell-based assay is shown to be more sensitive than fixed cell assay, especially for serum test^15^. However, whether ethnic disparity plays a part in the variability is under-investigated. A recent multi-center study from Europe, which reported the prevalence of anti-NMDA receptor antibodies in their cohort by ethnicity, showed marked variations in prevalence among the ethnic groups^8^. Thus, we hypothesize that there is a potential ethnic variation in the prevalence of anti-NMDA receptor antibodies among patients with psychosis, and further studies are needed to test this hypothesis.

The limitations of the current study include the small sample size and its single-center design. While the study samples were consecutively recruited, the selection of patients undergoing the test for anti-NMDA receptor antibodies is biased by clinical suspicion, affecting the generalizability of the results. However, such bias is more likely to lead to an overestimation of prevalence, since the tested patients were more likely to have an acute onset, a key characteristic of autoimmune psychosis/encephalitis^10^. The age-related inclusion criteria, which are a restriction due to the scope of our team’s service, mean that potentially some cases of young-onset psychosis or encephalitis are missed from the study.

## Methods

This is a retrospective cross-sectional study. We reviewed all the cases being assessed by the consultation liaison psychiatry team at the Accident and Emergency Department or inpatient departments of is a major tertiary hospital of Hong Kong serving a catchment area of a population of 700 thousand people, for first-onset psychosis from January 2015 to March 2025. The inclusion criteria are: 1. Age 18-65 years old at the time of presentation; 2. Presenting with first-onset psychosis, regardless of etiologies; 3. Received test(s) for the presence of anti-NMDA receptor antibodies in serum or cerebrospinal fluid (CSF) sample. The presence of anti-NMDA receptor antibodies (IgG class against the NR1 subunit) was tested by cell-based indirect immunofluorescence assay qualitatively (by EUROIMMUN; using a 1:10 dilution for serum and no dilution for CSF samples) in a certified laboratory of another teaching hospital. The patients selected for autoantibodies testing were based on the treating clinician’s suspicion of autoimmune encephalitis, but there were no operational guidelines. Among the 426 cases screened, 37 were excluded as they did not have first-onset psychotic symptoms as assessed by the attending psychiatric team, and a further 280 patients were excluded as they did not receive anti-NMDA receptor testing (See supplementary figure). The prevalence is presented using summary statistics, and we applied independent t-tests, Fisher’s exact tests, and chi-square tests to compare the characteristics of patients with and without the anti-NMDAR test (Table 1). The study was approved by the Joint CUHK-NTEC CREC (ref no.: 2024.606).

## Supplementary information

Supplementary Figure

## Data Availability

The data are available on reasonable request.
