# Supplementary figures and images for: Prevalence of anti-NMDA receptor antibodies among adult patients presenting with first-episode psychosis in Hong Kong
Source: Schizophrenia (Heidelb). 2026 Feb 25;12(1):35. doi: 10.1038/s41537-026-00741-2 (PMC13057035; doi:10.1038/s41537-026-00741-2)

**Supplementary figure**

Recruitment flowchart


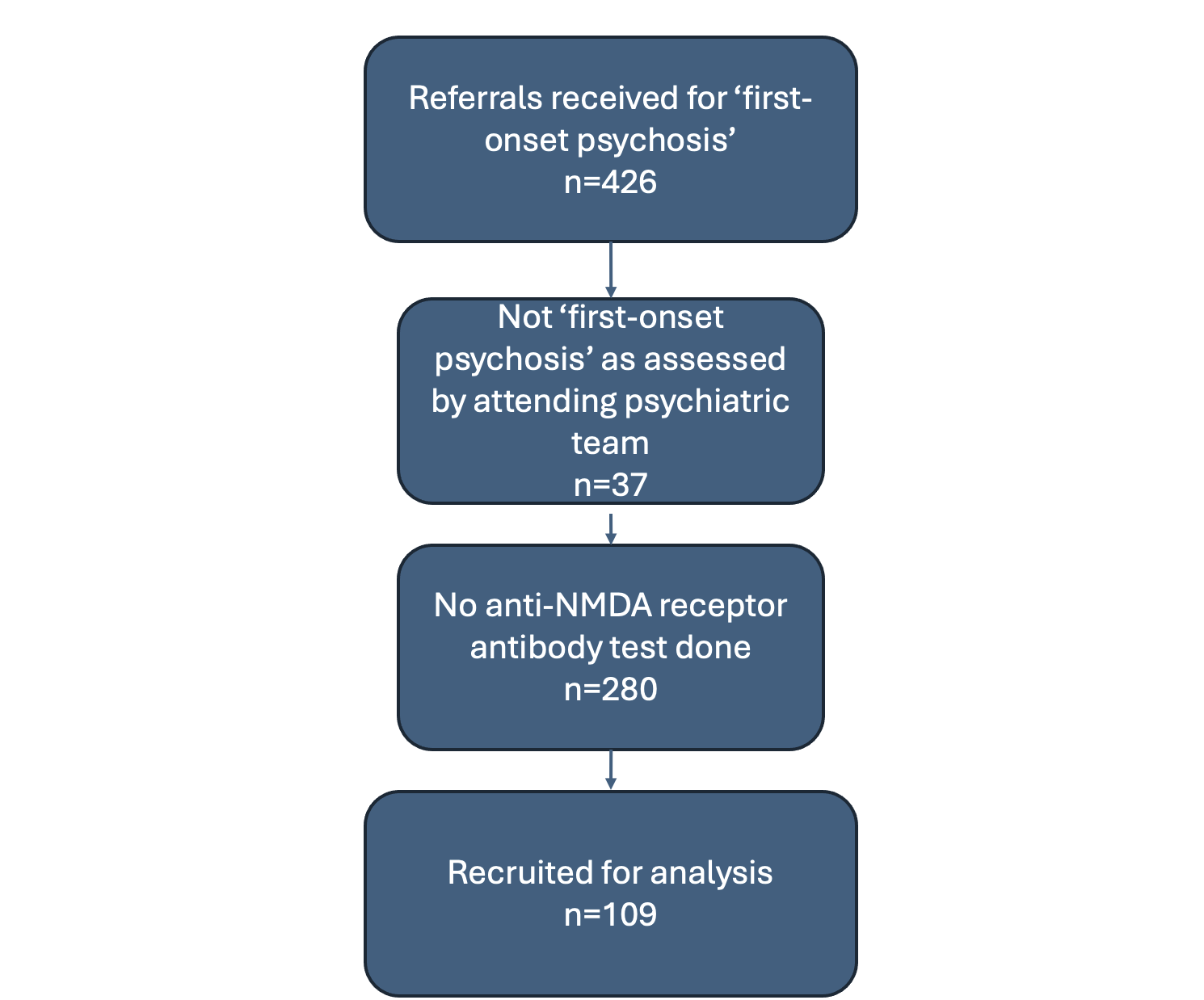

Supplement: Supplementary file 1 — Supplementary Figure [file 41537_2026_741_MOESM1_ESM.docx]
